# Supplementary material for: Geriatric Dentistry Curriculum in Six Continents
Source: Int J Environ Res Public Health. 2020 Jun 29;17(13):4682. doi: 10.3390/ijerph17134682 (PMC7370086; doi:10.3390/ijerph17134682)
Supplement: Supplementary file 1 [file ijerph-17-04682-s001.pdf]

International Journal of
Environmental Research and Public Health

Supplsupporting Information

Table S1. Curriculum content in geriatric dentistry in Dental Schools questionnaire.

**Curriculum content in geriatric dentistry in dental schools**
1. Name of dental school and country of origin?<br>__________________________
2. Is it a public or private school? Mark only one oval.<br>• Public<br>• Private
3. Years of predoctoral education at your institution? Mark only one oval.<br>• 3 year program<br>• 4 year program<br>• 5 year program<br>• 6 year program<br>• Other:__________________
4. Indicate the years of predoctoral education Geriatric Dentistry is taught:<br>_________________________________
5. Average size of graduating class? (Nº students)<br>_________________________________
6. Is expansion of geriatric teaching currently being planned? Mark only one oval.<br>• Yes<br>• No
7. Position of person answering the questionnaire? Mark only one oval.<br>• Dean or Administrative Officer<br>• Department Chairman<br>• Program Director for Geriatric Dentistry<br>• Other:____________________________
8. Didactic courses/seminars in geriatric dentistry is: Mark only one oval.<br>• Elective

- Compulsory

9. The clinical education program in geriatric dentistry teaching is: Mark only one oval.
- Elective
- Compulsory

10. The geriatric program consists of: Tick all that apply.
- Independent courses
- Organized series of lectures
- Occasional lectures
- Formal course
- Other:_____________________

11. If you have a geriatric program, who is responsible for teaching it? Mark only one oval.
- Director of program in geriatric dentistry.
- No person in particular.
- Multidisciplinary team.
- Other:________________________

12. The person responsible for teaching geriatric dentistry is: Tick all that apply.
- Formally trained in geriatric dentistry, geriatric or gerontology
- Trained in prosthodontics
- Trained in oral medicine
- Trained in working with special needs populations
- Trained in dental public health
- Other:____________________________

13. What is the main method by which didactic geriatric dentistry is presented to your students? Mark only one oval.
- A specific independent course in geriatric dentistry
- An organized series of presentations in other courses in the curriculum
- Occasional lectures as part of some of the clinical specialties
- No geriatric dentistry is taught
- Other:_____________________________

14. Inclusion of geriatric dentistry teaching into curriculum over time: Mark only one oval.
- Teaching geriatric dentistry
- As a required course
- As a specific course
- Lecture in another course

- Integration into curriculum
- Other:______________________________

15. Which departments lecture Geriatric Dentistry? Tick all that apply.

- Preventive, community, public health
- Prosthodontics
- Comprehensive Care, Family Dentistry
- Special patient care
- Multiple departments
- Oral surgery
- Other:________________________________

16. Which students participate in the didactic geriatric dentistry curriculum? (please check all that apply)

- 1st year dental students
- 2nd year dental students
- 3rd year dental students
- 4th year dental students
- 5th year dental students
- 6th year dental students

17. The format of teaching geriatric dentistry in the curriculum is: Mark only one oval.

- A didactic course or seminar only.
- A clinical rotation in extramural sites only.
- Combination of a and b.
- Other:____________________________

18. Which topics are covered in the curriculum? Mark only one oval per row.

Elective/ Compulsory

- Medical problems of the elderly
- Drug induced dental disease in the elderly
- Oral manifestations of systemic disease of the elderly Diagnosis and management of oral conditions of the elderly
- (caries, endodontics, periodontal disease, oral problems)
- Oral and dental tissue changes associated with aging Restorative management of the elderly using modifications of standard techniques
- Nutritional problems of the aging patient
- Demographic distribution of the elderly
- Socio-economic problems
- Psychosocial problems "Aging" and theories of aging Barriers to dental care Geriatric assessment scales
- Visual and auditory loss in the elderly
- Speech and hearing problems

- Oral management of the functionally independent adult Oral management of the frail elderly
- Oral management of the homebound, institutionalized and hospitalized elderly
- Home care and the use of portable equipment
- Restorative management of the elderly with neurological problems (Parkinson's, myasthenia graves, etc.)
- Restorative management of persons with depression Restorative management of persons with Alzheimer's disease and other dementia's
- Restorative management of the elderly with regards to adaptation and learning
- Other

19. If clinical care occurs at a remote site, where is it? Tick all that apply.
- Nursing home
- Geriatric hospital/ward
- Old people's home
- Geriatric day care center
- Congregate meals
- Clinic in a sheltered housing or high rise apartments
- Satellite or community clinics
- House calls
- Senior center
- Recreation center
- Churches
- Other:_________________________

20. If you have predoctoral curriculum in geriatric dentistry, who is responsible for coordinating it? Mark only one oval.
- Director of program in geriatric dentistry
- No one in particular
- Multidisciplinary team
- Other:______________________________

21. Which departments have the main responsibility for teaching the didactic course? Mark only one oval.
- Preventive, community, health ecology
- Prosthodontics
- Comprehensive care, family dentistry
- Special patient care
- Multiple departments
- Other:_____________________________________

22. Which departments have the main responsibility for teaching the clinical course? Mark only one oval.
- Preventive, community, health ecology
- Prosthodontics

- Comprehensive care, family dentistry
- Special patient care
- Multiple departments
- Other:______________________________

23. How is the geriatric program currently funded? Mark only one oval per row.

Undergraduate Program/ Postgraduate Program

- Federal government
- Local government
- Private grant
- Patient revenue (fee for service)
- As a special program with restricted funds

24. How many thesis, undergraduate degree work or publications on Gerontology, Geriatrics or Geriatric Dentistry have been made in the last 10 years?

____

25. Final thoughts?

____

Table S2. Inclusion of geriatric dentistry teaching into curriculum over time, expressed as percentage from total responses.

	**USA 1974[4,8,9]**	**USA 1979[4,8,9]**	**USA 1985[4,8,9]**	**Canada 1998[11]**	**USA 1998[4,8,9,11]**	**USA 2003[4,6,8]**	**Chile 2014[3]**	**USA 2016[4,9]**	**North America 2019**	**South America 2019**	**Europe 2019**	**Africa 2019**	**Asia 2019**	**Oceania 2019**
**Type of instruction**	N = 59	N = 58	N = 56	N = 9	N = 52	N = 54	N = 19	N = 56	N = 10	N =3 0	N = 24	N = 4	N = 13	N = 2
Teaching geriatric dentistry	57.6	100.0	100.0	33.0	100.0	100.0	84.0	100.0	100.0	100.0	100.0	100.0	100.0	100.0
As a required course	0.0	77.5	66.6	33.0	88.0	98.0	-	92.8	30.0	16.7	4.3	0.0	15.4	0.0
As a specific course	0.0	12.1	58.0	22.0	83.0	50.0	37.0	62.5	0.0	6.7	21.7	0.0	30.8	0.0
Lecture in another course	44.1	69.0	85.0	44.0	44.1	22.0	32.0	25.0	20.0	3.3	21.7	25.0	0.0	50.0
Integrated into curriculum	-	29.3	-	78.0	38.5	18.0	10.0	8.9	10.0	60.0	26.1	75.0	46.2	50.0
Other	-	5.2	0.0	-	13.5	8.0	-	3.6	30.0	0.0	0.0	0.0	0.0	0.0

Table S3. Comparison of dedicated gerodontology teaching formats in over 40 years, expressed as percentage from total responses.

	USA 1974[8]	USA 1979[8,13]	USA 1985[8]	USA 1998[8]	USA 2003[6,8]	Austria, Switzerland, Germany 2004[5]	Austria, Switzerland, Germany 2009[5]	Austria, Switzerland, Germany 2014[5]	USA 2016[4,10]	North America 2019	South America 2019	Europe 2019	Africa 2019	Asia 2019	Oceania 2019
**Comparison of formats**	N = 59	N = 58	N = 56	N = 52	N = 54	N = 38	N = 37	N = 33	N = 56	N = 10	N = 30	N = 24	N = 4	N = 13	N = 2
A didactic course or seminar	-	69.0	-	-	98.0	10.5	13.5	9.1	-	10.0	23.3	33.3	25.0	38.5	0.0
A clinical rotation in extramural sites	43.0	78.0	64.0	55.0	67.0	7.9	13.5	6.0	67.9	0.0	6.7	4.2	0.0	0.0	0.0
Combination of both	-	-	-	-	28.0	15.8	21.6	18.2	-	90.0	60.0	54.2	50.0	53.8	100.0
Other	-	-	-	-	9.0	-	-	-	-	0.0	10.0	8.3	25.0	7.7	0.0

Table S4. Topics covered by lectures and seminars: (A) traditional topics (oral medicine/prosthodontics) and (B) non-traditional topics (gerontologic), expressed as a percentage from total responses.

(A) Traditional Topics taught over the past 30 y.	USA 1979[13]	USA 1981[9]	USA 1987[9]	USA 1998[9]	USA 2003[6,9]	Europe 2005[12]	Chile 2014[3]	USA 2015[4]		Europe 2016[2]	USA 2016[9]		Six continents 2019	
								Compulsory	Elective		Compulsory	Elective	Compulsory	Elective
	N = 58	N = 58	N = 56	N = 52	N = 54	N = 82	N = 19	N = 56	N = 56	N = 123	N = 53	N = 3	N = 83	N = 83
Medical problems of the elderly	81.0	81.0	100.0	93.7	100.0	84.0	100.0	91.1	3.6	70.7	96.2	66.7	67.9	32.1
Oral and dental tissue changes associated with aging	79.3	79.3	100.0	92.1	98.0	83.0	85.0	83.9	3.6	61.0	89.3	66.7	66.3	33.7
Drug-induced dental disease in the elderly		-	100.0	92.1	-	-	-	85.7	3.6	-	94.3	100.0	65.3	34.7
Diagnosis and management of oral conditions of the elderly caries, endodontics, periodontal disease, oral problems	-	-	-	85.7	91.0	85.0	85.0	85.7	3.6	49.6	90.6	66.7	75.0	25.0
Oral manifestations of systemic disease of the elderly	84.5	84.5	100.0	58.7	96.0	87.0	85.0	89.3	5.4	-	88.6	66.7	73.1	26.9
Restorative management of the elderly using modifications of standard techniques	37.9	37.9	100.0	73.0	65.0	57.0	75.0	73.2	1.8	-	77.4	33.3	68.0	32.0
Nutritional problems of the aging patient	-	-	89.0	-	79.0	59.0	75.0	64.3	3.6	56.1	67.9	66.7	62.3	37.7

**(B) Non-traditional Topics Gerontologic topics taught over the past 30 y.**														
Barriers to dental care	48.3	48.3	95.0	-	100.0	54.0	70.0	94.6	3.6	45.5	100.0	66.7	68.6	31.4
Socioeconomic problems	72.4	72.4	100.0	87.3	92.0	56.0	85.0	92.9	5.4	-	98.1	100.0	65.1	34.9
Demographic distribution of the elderly	48.3	48.3	96.0	95.2	85.0	65.0	100.0	92.9	5.4	61.8	98.1	100.0	67.7	32.3
Psycho-social problems	75.9	75.9	-	87.3	98.0	66.0	85.0	83.9	5.4	-	88.6	100.0	59.1	40.9
Restorative management of persons with Alzheimer's disease and other dementias	-	37.9	90.0	68.3	-	-	-	80.4	1.8	-	84.9	33.3	61.7	38.3
"Ageing" and theories of ageing	58.6	58.6	81.0	-	85.0	61.0	100.0	78.6	3.6	57.7	83.0	66.7	58.8	41.2
Oral management of the functionally independent adult	-	72.4	78.0	84.1	91.0	72.0	85.0	78.6	3.6	-	83.0	66.7	68.2	31.8
Oral management of the frail elderly	44.8	44.8	80.0	84.1	77.0	56.0	75.0	78.6	5.4	48..0	83.0	100.0	59.1	40.9
Oral management of the homebound, institutionalized and hospitalized elderly	44.8	44.8	80.0	84.1	59.0	48.0	55.0	73.2	3.6	-	77.4	66.7	58.7	41.3
Visual and auditory loss in the elderly	32.8	32.8	-	-	68.0	39.0	75.0	71.4	3.6	-	75.5	66.7	63.2	36.8
Speech and hearing problems	-	-	-	-	-	-	-	69.6	3.6	-	73.6	66.7	58.5	41.5
Restorative management of the elderly with neurological problems Parkinson's, myasthenia graves, etc.	37.9	37.9	-	68.3	62.0	54.0	55.0	67.9	1.8	-	71.7	33.3	54.7	45.3
Geriatric assesment scales	-	-	-	-	60.0	23.0	70.0	78.6	5.4	47.2	64.2	66.7	50.9	49.1
Restorative management of the elderly with regards to adaptation and learning	41.4	37.9	78.0	-	60.0	63.0	85.0	57.1	3.6	-	60.4	66.7	59.6	40.4
Home care and the use of portable equipment	-	-	-	-	-	32.0	30.0	-	-	28.5	-	-	50.9	49.1
Other	8.6	-	-	-	-	-	30.0	19.6	1.8	-	-	-	59.1	40.9

Table S5. Funding of geriatric undergraduate programs, expressed as percentage from total responses.

	**USA 1979[8,13]**	**USA 1985[8]**	**USA 1998[8]**	**USA 2003[8]**	**North America 2019**	**South America 2019**	**Europe 2019**	**Africa 2019**	**Asia 2019**
Source of funds	N = 58	N = 56	N = 52	N = 54	N = 6	N = 23	N = 18	N = 2	N = 8
Line item in school budget	0.0	-	58.1	40.0	-	-	-	-	-
Federal government	9.8	10.7	9.7	1.0	0.0	52.2	88.9	50.0	25.0
Local/ state government	26.4	53.5	8.1	7.0	33.3	34.8	27.8	50.0	25.0
Corporate/private grants	5.5	-	9.7	0.0	33.3	13.0	22.2	0.0	12.5
Patient revenue	44.4	79.0	38.7	52.0	66.7	13.0	33.3	50.0	25.0
As a special program with restricted funds	-	-	-	-	33.3	17.4	11.1	0.0	12.5
Other	13.9	-	17.7	-	-	-	-	-	-

Table S6. Remote sites of clinical geriatric care, expressed as percentage from total responses.

	**USA 1974[8]**	**USA 1979[8,13]**	**USA 1985[8]**	**USA 1998[8]**	**USA 2016[4,10]**	**North America 2019**	**South America 2019**	**Europe 2019**	**Africa 2019**	**Asia 2019**	
**Sites**	N = 59	N = 58	N = 56	N = 52	N = 56	N = 8	N = 18	N = 13	N = 1	N = 10	N = 1
Nursing home	39.0	43.1	27.0	55.0	37.5	75.0	44.4	46.2	0.0	0.0	100.0
Geriatric hospital/ ward	15.3	17.2	40.0	20.0	0.0	50.0	27.8	30.8	0.0	0.0	100.0
Old people's home	-	-	-	-	-	25.0	50.0	38.5	100.0	70.0	100.0
Geriatric day care center	-	15.5	-	13.0	14.3	25.0	55.6	23.1	0.0	0.0	100.0
Congregate meals	-	-	-	-	-	0.0	5.6	0.0	0.0	0.0	0.0
Clinic in a sheltered housing or high-rise apartments	8.5	6.9	-	12.0	-	12.5	5.6	0.0	0.0	0.0	
Satellite or community clinics	20.3	31.0	-	18.0	60.7	37.5	0.0	23.1	100.0	70.0	100.0
House calls	-	-	-	-	-	25.0	5.6	15.4	0.0	0.0	0.0
Senior center	-	-	-	-	21.4	50.0	11.1	7.7	0.0	0.0	
Recreation center	-	-	-	-	-	12.5	5.6	0.0	0.0	0.0	0.0
Churches	-	-	-	-	7.1	0.0	11.1	0.0	0.0	0.0	0.0
Mobile Unit	10.2	10.3	1.8	8.0	12.5	0.0	0.0	0.0	0.0	0.0	0.0

Table S7. Department responsible for teaching didactic (D) and clinical (C) geriatric dentistry, expressed as percentage from total responses.

	**USA 1976[13]**	**USA 1979[13]**	**USA 2016[4]**		**Austria 2017[5]**	**Switzer-land 2017[5]**	Germany 2017[5]	North America 2019		South America 2019		Europe 2019		Africa 2019		Asia 2019		Oceania 2019	
**Department**	**N = 34**	**N = 58**	**D N = 55**	**C N = 55**	**N = 7**	**N = 6**	N = 53	D N = 10	C N = 10	D N = 29	C N = 28	D N = 21	C N = 21	D N = 4	C N = 4	D N = 12	C N = 12	D N = 2	C N = 2
Preventive, community, health ecology	70.6	51.7	28.6	25.0	43.8	35.3	50.0	0.0	0.0	13.8	17.9	19.0	9.5	25.0	25.0	0.0	0.0	0.0	0.0
Prosthodontics	32.4	58.6	5.4	5.4	75.0	25.0	60.0	10.0	20.0	31.0	35.7	28.9	42.9	25.0	25.0	25.0	33.3	0.0	0.0
Comprehensive care, family dentistry	11.8	6.9	17.9	17.9	50.0	25.0	55.0	30.0	40.0	0.0	0.0	0.0	0.0	0.0	0.0	0.0	8.3	0.0	0.0
Special patient care	5.9	13.8	7.1	7.1	-	-	-	0.0	0.0	3.4	0.0	9.5	14.3	25.0	25.0	0.0	0.0	100.0	100.0
Multiple departments	5.9	19.0	21.4	21.4	-	-	-	50.0	30.0	41.4	35.7	28.9	23.8	0.0	25.0	75.0	58.3	100.0	100.0
Oral medicine	5.9	10.3	-	-	25.0	25.0	40.0	0.0	0.0	0.0	0.0	0.0	0.0	0.0	0.0	0.0	0.0	0.0	0.0
Other	20.0	-	17.9	21.4	-	-	-	10.0	10.0	10.3	10.7	14.3	9.5	25.0	25.0	0.0	0.0	0.0	0.0
